# A Kinome Screen Identifies Checkpoint Kinase 1 (CHK1) as a Sensitizer for RRM1-Dependent Gemcitabine Efficacy

**DOI:** 10.1371/journal.pone.0058091

**Published:** 2013-03-04

**Authors:** Jun Zhou, Zhengming Chen, Agnes Malysa, Xin Li, Paula Oliveira, Yingtao Zhang, Gerold Bepler

**Affiliations:** Molecular Therapeutics Program and Molecular Imaging and Biomarkers Program, Karmanos Cancer Institute, Detroit, Michigan, United States of America; Vanderbilt University Medical Center, United States of America

## Abstract

Gemcitabine is among the most efficacious and widely used antimetabolite agents. Its molecular targets are ribonucleotide reductase M1 (RRM1) and elongating DNA. Acquired and *de novo* resistance as a result of RRM1 overexpression are major obstacles to therapeutic efficacy. We deployed a synthetic lethality screen to investigate if knockdown of 87 selected protein kinases by siRNA could overcome RRM1-dependent gemcitabine resistance in high and low RRM1-expressing model systems. The models included genetically RRM1-modified lung and breast cancer cell lines, cell lines with gemcitabine-induced RRM1 overexpression, and a series of naturally gemcitabine-resistant cell lines. Lead molecular targets were validated by determination of differential gemcitabine activity using cell lines with and without target knock down, and by assessing synergistic activity between gemcitabine and an inhibitor of the lead target. CHK1 was identified has the kinase with the most significant and robust interaction, and it was validated using AZD7762, a small-molecule ATP-competitive inhibitor of CHK1 activation. Synergism between CHK1 inhibition and RRM1-dependent gemcitabine efficacy was observed in cells with high RRM1 levels, while antagonism was observed in cells with low RRM1 levels. In addition, four cell lines with natural gemcitabine resistance demonstrated improved gemcitabine efficacy after CHK1 inhibition. In tumor specimens from 187 patients with non-small-cell lung cancer, total CHK1 and RRM1 *in situ* protein levels were significantly (*p* = 0.003) and inversely correlated. We conclude that inhibition of CHK1 may have its greatest clinical utility in malignancies where gemcitabine resistance is a result of elevated RRM1 levels. We also conclude that CHK1 inhibition in tumors with low RRM1 levels may be detrimental to gemcitabine efficacy.

## Introduction

Gemcitabine is one of the most widely used agents with proven efficacy in non-small-cell lung cancer (NSCLC), breast cancer, pancreatic cancer, and bladder cancer among others [1], [2], [3], [4]. *De novo* and acquired resistance, however, limit its utility to a subset of patients and constrain efficacy to a period of time measured in months.

Gemcitabine’s mechanism of action has been extensively studied, and the two major molecular mechanisms are inhibition of deoxynucleotide synthesis and blockade of DNA synthesis [5], [6], [7], [8]. The former is achieved through covalent binding to and inactivation of the regulatory subunit of ribonucleotide reductase M1 (RRM1). The latter is achieved through integration into the nascent DNA, which results in an elongation block.

In cell lines and animal models, emergence of gemcitabine resistance coincides with increased RRM1 expression [9], [10], and gemcitabine efficacy can be influenced directly through modulation of RRM1 levels [11], [12]. RRM1 has been identified as the major determinant of gemcitabine efficacy in patients treated with this agent. High levels of tumoral RRM1 are predictive of treatment resistance while low levels are associated with treatment efficacy [11], [12], [13], [14]. In addition, limited data suggest that RRM1 levels can rise in patients receiving gemcitabine-based therapy [15].

Increasing gemcitabine efficacy in patients with *de novo* resistance and reversing or delaying acquired resistance would be major therapeutic advances. Our prior investigations had identified a link between RRM1 and signal transduction pathways [16], [17] as well as G2 cell cycle arrest [18]. Here, we investigated the impact of selected kinases involved in cellular proliferation and cell cycle control on the RRM1-modulated gemcitabine efficacy using synthetic lethal screens. We identified CHK1 as a major target capable of reversing gemcitabine resistance.

## Results

### Knockdown of Selected Protein Kinases Impacts RRM1-dependent Cell Viability

To assess the interaction of selected kinases with RRM1, and ultimately a combined gene modification on gemcitabine efficacy, a synthetic lethality screen was performed using transfection of siRNAs specific to each of 87 different receptor and non-receptor tyrosine and serine/threonine kinases critical for cell cycle, shape, proliferation, and differentiation control. Non-targeting siRNA was used as a control, and cell viability was used as a read-out.

**Figure 1:**
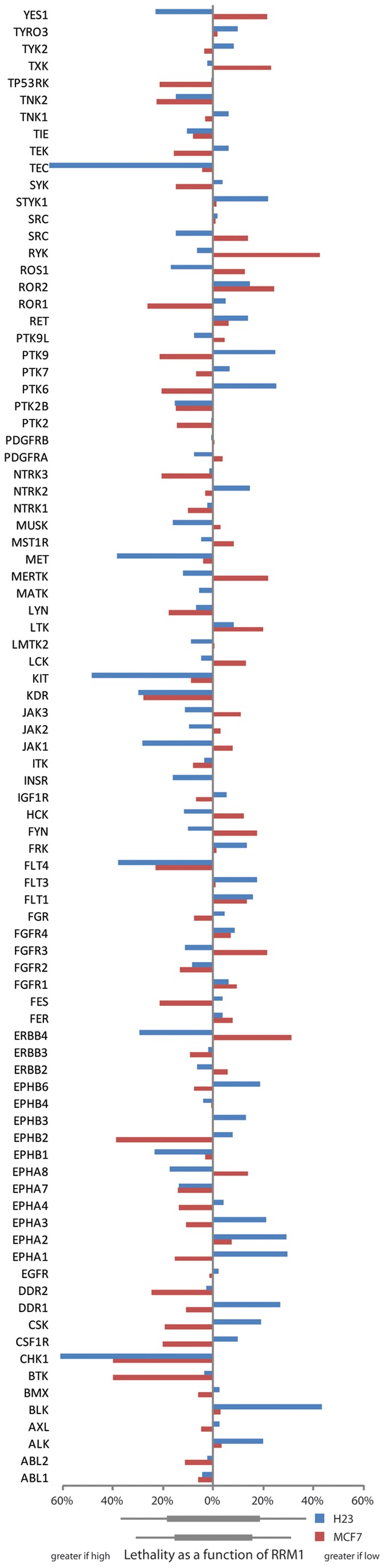
Synthetic lethality of 87 kinases as a function of RRM1 expression. Two cell line systems, H23 (blue bars) and MCF7 (red bars), with stably increased or reduced RRM1 expression were transfected with gene-specific siRNAs and viability was scored. Bars to the left indicate greater lethality in high compared to low RRM1-expressing cells. Bars to the right indicate greater lethality in low compared to high RRM1-expressing cells. Bars below the x-axis indicate one (wide bar) or two (narrow bar) standard deviations in the lethality scores for the two cell line systems respectively.

Screens were performed in 96-well plates using two replicates with siGENOME SMARTpool siRNA in each of the four sublines of the H23 and MCF7 stably RRM1-modified model systems (H23-R1, H23-shR1, H23-Ct, H23-wt, and MCF7-R1, MCF7-shR1, MCF7-Ct, MCF7-wt) without gemcitabine. Differential cell viability was calculated comparing values obtained from high (R1) and low (shR1) RRM1 expressers (Figure 1). In the H23 system, knockdown of the genes CHK1, FLT4, KIT, MET, and TEC had a significantly (*p≤*0.01) greater lethality if RRM1 expression was high, and BLK had a significantly greater lethality if RRM1 expression was low. In the MCF7 system, knockdown of the genes BTK, CHK1, and EPHB2 had a significantly (*p≤*0.01) greater lethality if RRM1 expression was high, and ERBB4 and RYK had a significantly (*p≤*0.01) greater lethality if RRM1 expression was low. In addition, in both cell line systems knockdown of the gene KDR had a significantly (*p≤*0.05) greater lethality if RRM1 expression was high.

We attempted to verify these results with ON-TARGET*plus* SMARTpool siRNA using four separate replicate experiments. Only the genes CHK1, KDR, and TEC yielded similar results with significantly (*p≤*0.05) greater lethality if RRM1 expression was high in both cell line systems. BLK yielded similar results for the H23 system only with significantly (*p≤*0.05) greater lethality if RRM1 expression was low.

### Knockdown of Selected Kinases Modulates RRM1-dependent Gemcitabine Efficacy

BLK, CHK1, KDR, KIT, MET, and TEC were chosen to study interactions with RRM1-dependent gemcitabine efficacy. BLK, KDR, KIT, and MET knockdown did not appreciably impact gemcitabine efficacy, and both had no interaction with RRM1 in the H23 model (Table 1); i.e., there was no differential activity in high versus low RRM1 expressing cells (IC50 ratios of 0.79, 1.10, 0.85, and 1.02 respectively for high vs. low RRM1).

**Table 1:** Interaction of selected kinases with RRM1-dependent gemcitabine efficacy.

IC50 ratio*	BLK	CHK1	KDR	KIT	MET	TEC	IC50 in siRNAControl [nM]
H23(control)	1.20	0.55	0.82	0.96	1.13	0.64	6.99
H23-R1(RRM1 high)	1.10	0.26	1.39	0.84	1.19	0.74	11.73
H23-shR1(RRM1 low)	1.39	3.80	1.26	0.99	1.17	1.24	4.68
MCF7(control)		0.45				0.68	3.06
MCF7-R1(RRM1 high)		0.18				0.67	58.58
MCF7-shR1(RRM1 low)		1.40				0.72	1.09
H125		0.76				0.94	8.20×10^3^
H650		0.02				0.95	47.12×10^3^
H1648		0.49				0.91	10.11×10^3^
H2122		0.29				1.00	54.54×10^3^
H23-G-C8		0.28					40.67
H23-G-C23		0.31					41.95
H1299-G-C2		0.28					243.07
H1299-G-C18		0.28					248.06

* The IC50 ratio was calculated by dividing the siRNA-specific IC50 by the control IC50. Cell lines H23, MCF7, and H1299 were exposed to gemcitabine for four 4 days. H125, H650, H1648, and H2122 were exposed for 5 to 7 days (a 4-day exposure yields IC50 values above 1 mM). RRM1 knockdown in the gemcitabine resistant clones restored sensitivity (IC50 ratios: 0.15 for H23-G-C8 and H23-G-C23, 0.19 for H1299-G-C2 and 0.09 for H1299-G-C18).

**Figure 2:**
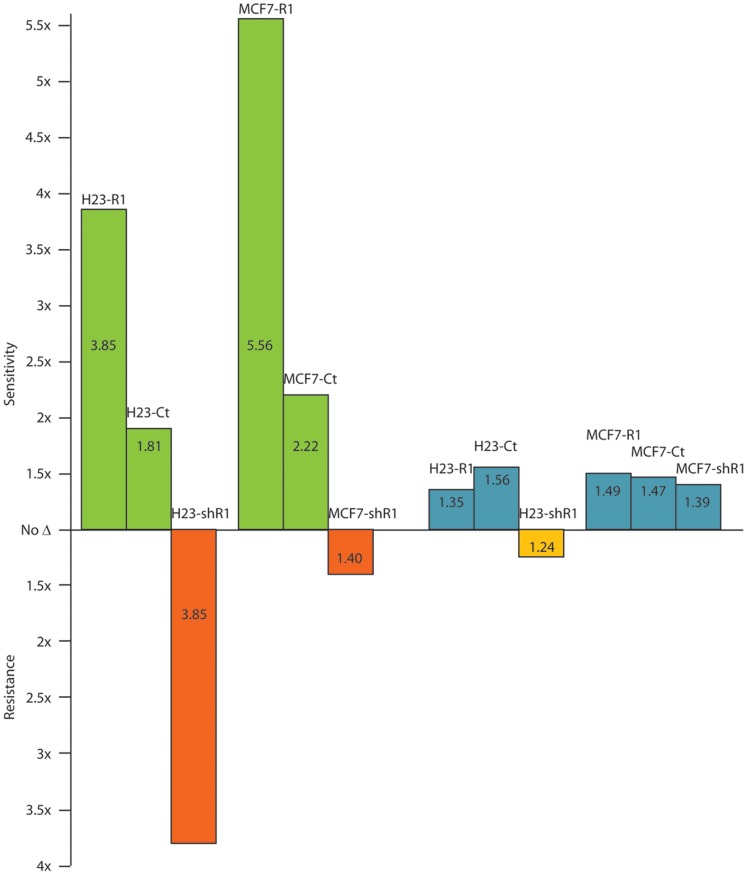
CHK1 and TEC knockdown and RRM1-dependent gemcitabine sensitivity. The H23 and MCF7 cell line systems with increased (−R1) and reduced (−shR1) RRM1 expression were transfected with CHK1-specific or TEC-specific siRNA and exposed to gemcitabine. IC50 values are the mean of four replicates. CHK1 is depicted in green and red, TEC is depicted in blue and orange.

**Figure 3:**
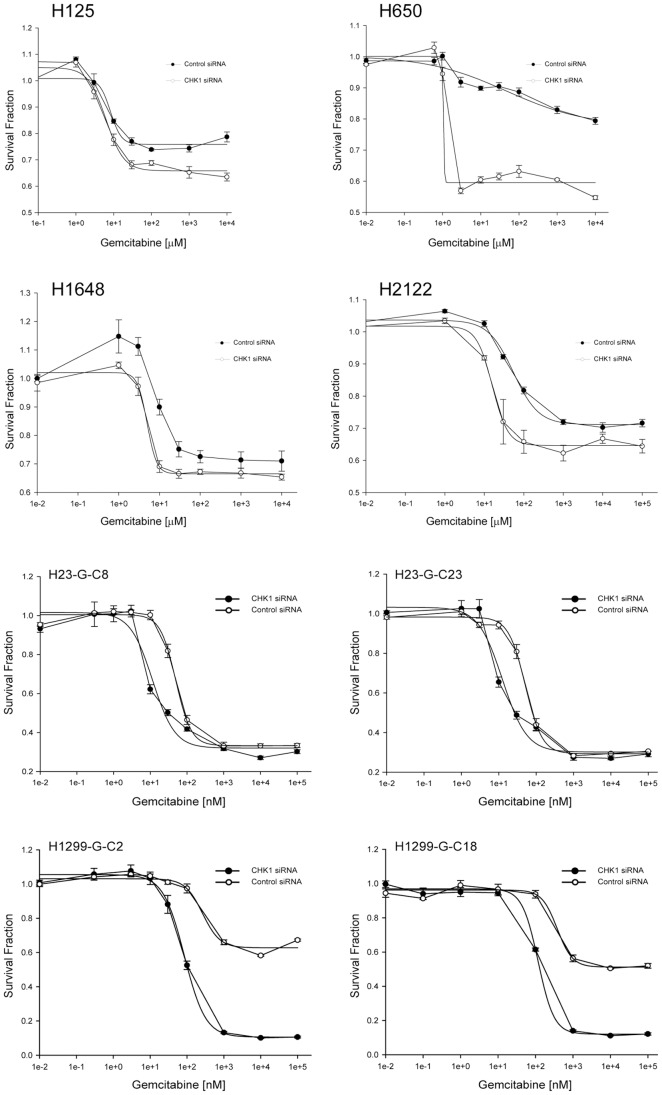
CHK1 knockdown can overcome gemcitabine resistance. Four naturally gemcitabine-resistant NSCLC cell lines (H125, H650, H1648, H2122) and four gemcitabine-resistant clones generated *in vitro* (H23-G-C8, H23-G-C23, H1299-G-C2, H1299-G-C18) were exposed to gemcitabine for 5 days. Data points are the mean of four replicates, and bars depict the standard error. There is a shift of the dose-response curve to the left in addition to an increase in the maximally achievable cytotoxicity.

CHK1 knockdown improved gemcitabine efficacy (1.8-fold improvement in IC50 concentration in H23 and 2.2-fold improvement in MCF7; Table 1), and it displayed a clear interaction with RRM1 in both models. The gemcitabine IC50 improved at least 3.8-fold in cells with high RRM1 expression, and it deteriorated 1.4 to 3.8-fold in cells with low RRM1 expression (Table 1; Figure 2). CHK1 knockdown also substantially improved gemcitabine efficacy in four naturally gemcitabine-resistant NSCLC cell lines (H125, H650, H1648, H2122 [19]) with a shift of the dose-response curve to the left in addition to an increase in the maximally achievable cytotoxicity (IC50 ratios of 0.02 to 0.76; Figure 3; Table 1). Finally, we generated gemcitabine resistant clones from cell lines H23 and H1299 *in vitro* by prolonged drug exposure. In these clones, gemcitabine resistance was associated with a 20-fold increase in RRM1 mRNA and protein expression; and RRM1 knockdown with ON-TARGET*plus* SMARTpool siRNA restored gemcitabine sensitivity (IC50 ratios: 0.15 for H23-G-C8 and H23-G-C23, 0.19 for H1299-G-C2 and 0.09 for H1299-G-C18). In this model system, CHK1 knockdown reversed gemcitabine resistance and resulted in a near 4-fold improvement in drug efficacy (Figure 3; Table 1).

TEC knockdown minimally improved gemcitabine efficacy (1.5-fold improvement in IC50 concentration) and displayed an interaction with RRM1 (Table 1; Figure 2). TEC knockdown slightly improved gemcitabine efficacy in H23 with high RRM1 expression and reduced efficacy in cells with low RRM1 expression. This interaction was not observed in the MCF7 model. We further investigated the impact of TEC knockdown on gemcitabine efficacy in the four NSCLC cell lines with wild-type resistance to gemcitabine, and no evidence for altered gemcitabine sensitivity was observed in these cell lines (Table 1).

### A Small-molecule CHK1 Inhibitor Increases Gemcitabine Efficiency

**Figure 4:**
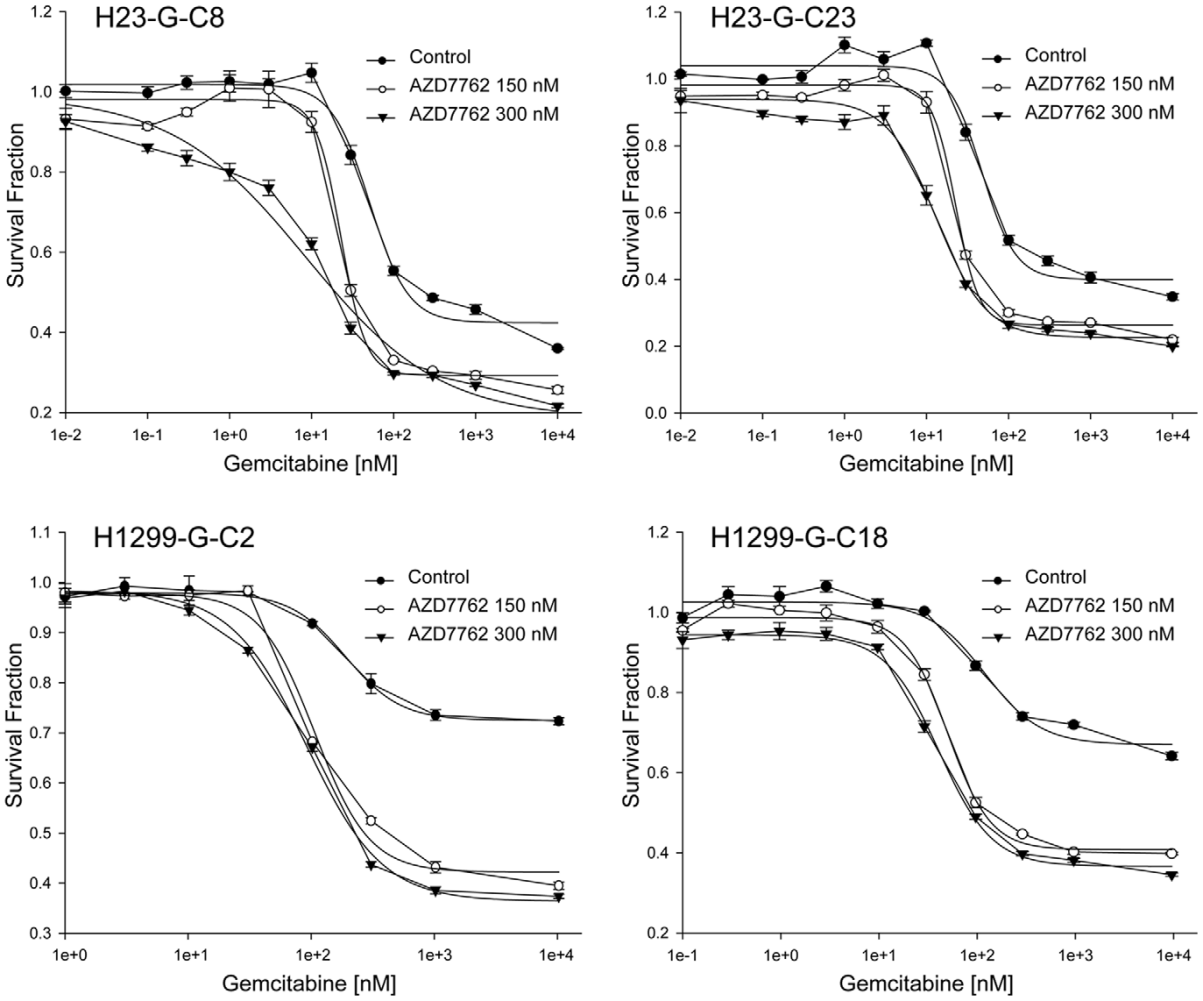
The CHK1 inhibitor AZD7762 increases gemcitabine efficacy. Four gemcitabine-resistant clones were exposed to drugs for 4 days. Data points are the mean of four replicates, and bars depict the standard error. The IC50 values were as follows: For clone H23-G-C8, control 51.4 nM, AZD7762-150 nM 22.8 nM, AZD7762-300 nM 8.5 nM; for clone H23-G-C23, control 47.7 nM, AZD7762-150 nM 22.6 nM, AZD7762-300 nM 13.2 nM; for clone H1299-G-C2, control 186.1 nM, AZD7762-150 nM 104.8 nM, AZD7762-300 nM 90.1 nM; for clone H1299-G-C18, control 123.8 nM, AZD7762-150 nM 52.0 nM, AZD7762-300 nM 42.0 nM.

We used AZD7762, a small-molecule ATP-competitive inhibitor of CHK1 activation [20], to study its impact on gemcitabine efficacy. The single-agent IC50 for AZD7762 was determined over a concentration range of 10 pM to 100 µM using 4-day exposure in the four gemcitabine-resistant clones of cell lines H23 and H1299, and it was 505 nM for H23-G-C8, 497 nM for H23-G-C23, 2.5 µM for H1299-G-C2, and 1.8 µM for H1299-G-C18. Cells were then exposed to increasing concentrations (10 pM–100 µM) of gemcitabine in the presence or absence of 150 nM or 300 nM AZD7762. All four clones showed an AZD7762 dose dependent increase in gemcitabine sensitivity (Figure 4). Two additional wild-type NSCLC cell lines (H1648, H2122) were investigated for efficacy modulation using the combination index (CI) methodology [21]. We observed clear synergism between both drugs in these gemcitabine-resistant model systems (Table 2).

**Table 2:** Gemcitabine and CHK1 inhibitor (AZD7762) combination index values in gemcitabine-resistant cell lines and clones.

Gem/AZD = 1/10	ED25	ED50	ED75	Dm	M	r
H1648	0.54 (synergism)	0.37 (synergism)	0.26 (strong synergism)	27.25	1.16	0.91
H2122	0.001 (very strong synergism)	0.008 (very strong synergism)	0.12 (strong synergism)	0.82	0.24	0.91
H23-G-C8	0.79 (moderate synergism)	0.73 (moderate synergism)	0.67 (synergism)	43.62	1.79	0.95
H23-G-C23	0.54 (synergism)	0.66 (synergism)	0.79 (moderate synergism)	32.91	1.24	0.95
H1299-G-C2	0.36 (synergism)	0.47 (synergism)	0.70 (moderate synergism)	76.52	1.19	0.98
H1299-G-C18	0.15 (strong synergism)	0.39 (synergism)	1.01 (nearly additive)	25.57	0.70	0.97

We also used AZD6474 (vandetanib), an oral inhibitor of KDR (VEGFR), EGFR, and RET [22], to study its impact on gemcitabine efficacy. The single-agent IC50 in the H23 and MCF7 model systems was between 10 and 17 µM without evidence for an RRM1-dependent activity differential.

### Knockdown of CHK1 and Gene Expression

**Figure 5:**
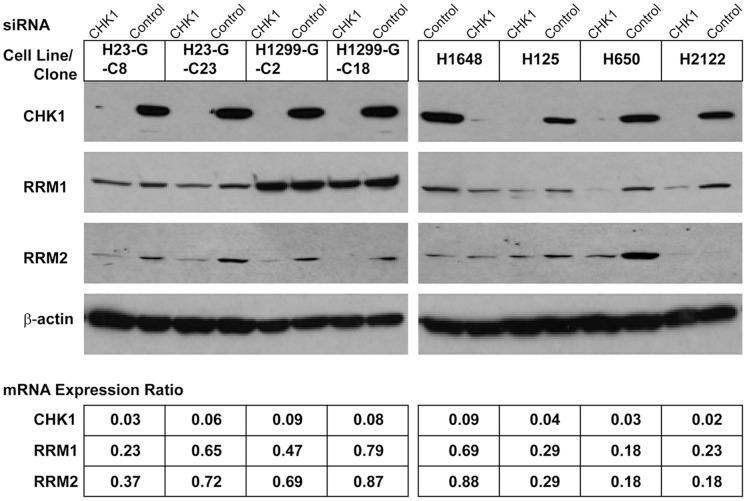
Protein and mRNA expression after CHK1 knowdown. CHK1 was knocked down using 30 nM ON-TARGET*plus* SMARTpool siRNA in four gemcitabine-resistant clones and four naturally gemcitabine-resistant cell lines. The mRNA expression ratios were calculated by dividing the gene expression values of CHK1 siRNA transfected cells by those of control siRNA transfected cells. No change in the expression levels of the genes *PTEN* and *MCM7* was observed.

To gain insight into the mechanism of the CHK1-mediated gemcitabine sensitization, we determined mRNA and protein expression levels for the genes *RRM1* and *RRM2* in the four naturally gemcitabine-resistant NSCLC cell lines and the four *in vitro*-induced gemcitabine-resistant clones 24 h after transfection. As depicted in Figure 5, transfection of cells with 30 nM CHK1 siRNA resulted in a 90% or greater reduction of CHK1 mRNA and protein and a 20–80% reduction in RRM1 and RRM2 mRNA and protein expression levels. To reduce concentration-dependent off-target effects, 10 nM concentrations of siRNA were used and the associated gene expression reductions were similar to those observed with 30 nM concentrations. We also assessed the expression levels of the genes *PTEN* and *MCM7*, and no changes were observed. Treatment of cells with 50–300 nM AZD7762 for up to 48 h did not result in a reduction of RRM1, RRM2, PTEN, or CHK1 levels.

In contrast, RRM1 knockdown with 10 nM or 30 nM RRM1 siRNA, which resulted in a 90% reduction of RRM1 protein levels, did not result in a reduction of CHK1 or RRM2 levels, but it did reduce PTEN levels as previously reported [16].

### Association of RRM1 and CHK1 *in situ* Protein Levels

**Figure 6:**
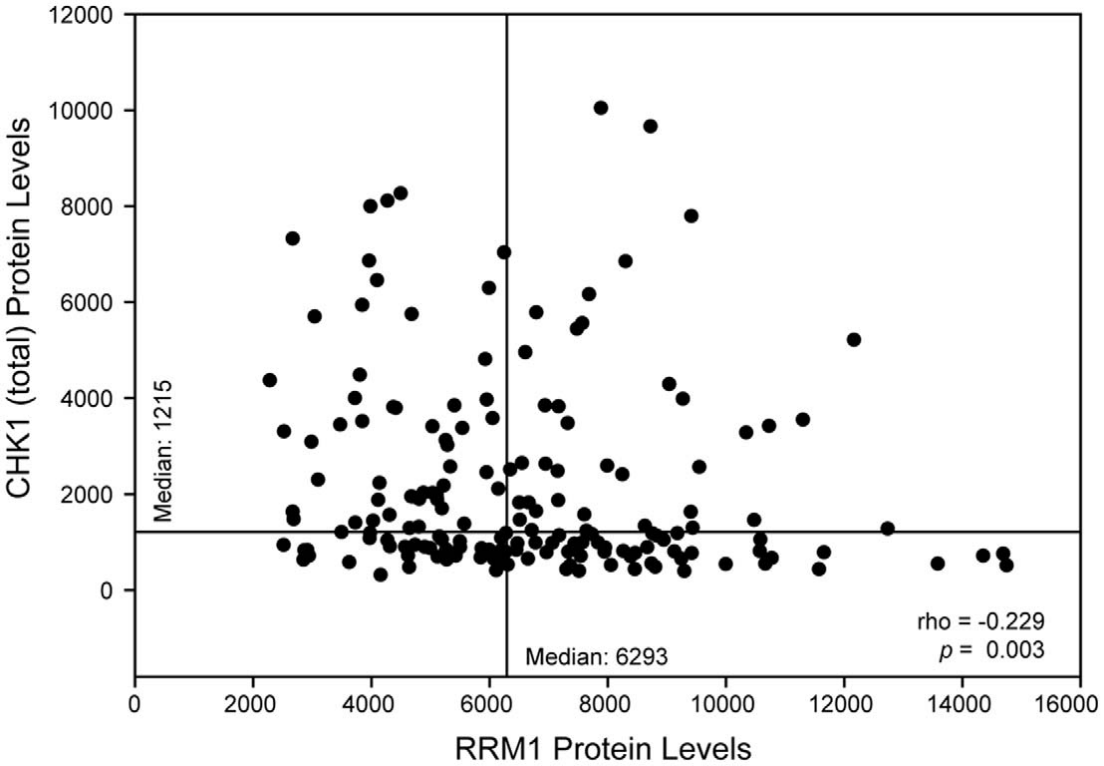
Correlation between total CHK1 and RRM1 protein levels. Protein levels in the nuclear compartment were determined by AQUA in three tumor replicates from 187 patients with lung cancer. The average values were plotted. Horizontal and vertical lines indicate the median levels for CHK1 and RRM1 respectively.

To investigate the relationships between RRM1 and total and phosphorylated CHK1 in human tumor samples from patients with NSCLC, we used a tissue microarray (TMA) consisting of 3 replicates of 187 surgically resected patients with stage I disease [23]. An immunofluorescence-based automated quantitative analysis (AQUA) system was used to measure nuclear *in situ* levels of RRM1, total CHK1, pCHK1(S280), pCHK1(S296), pCHK1(S317), and pCHK1(S345) [24]. Expression levels are summarized in table 3. Total CHK1 levels tended to decrease with increasing RRM1 levels (Figure 6); i.e., they were inversely correlated (r = −0.229, *p* = 0.003). pCHK1(S280), pCHK1(S296), and pCHK1(S317) levels tended to increase with increasing RRM1 levels (r = 0.155, *p = *0.043 for S280, r = 0.198, *p = *0.009 for S296, r = 0.366, *p*<0.001 for S317). pCHK1(S345) and RRM1 levels were not correlated.

**Table 3:** Total and phosphorylated CHK1 and RRM1 *in situ* protein levels.

	Chk1 (total)	pChk1(S280)	pChk1(S296)	pChk1(S317)	pChk1(S345)	RRM1
**All Cases,** Median (Range)*						
Median	1,215	210	187	116	267	6,293
Range	293–10,019	119–421	98–388	66–293	64–2,716	2,304–14,770
**Tumor Histology,** Mean (N)						
Adenocarcinoma	1,158 (96)	204 (96)	187 (96)	118 (96)	278 (96)	6,625 (88)
Squamous cell	1,325 (68)	214 (68)	190 (68)	115 (68)	251 (68)	5,990 (64)
Large cell	1,249 (22)	218 (22)	181 (22)	109 (22)	255 (22)	6,733 (21)
*p* - value	0.311	0.689	0.587	0.290	0.810	0.260
**Tumor Size,** Mean (N)						
≤2 cm	955 (60)	217 (60)	187 (60)	121 (60)	260 (60)	6,613 (54)
>2 - *≤*3 cm	1,206 (44)	201 (44)	196 (44)	119 (44)	261 (44)	6,328 (43)
>3 - *≤*5 cm	1,439 (55)	207 (55)	186 (55)	109 (55)	267 (55)	5,786 (50)
>5 - *≤*7 cm	996 (18)	206 (18)	195 (18)	110 (18)	296 (18)	6,090 (17)
>7 cm	2,274 (9)	210 (9)	181 (9)	117 (9)	268 (9)	5,948 (9)
*p* - value	0.129	0.765	0.772	0.190	0.947	0.144
**Smoking Status,** Mean (N)						
Never	961 (11)	197 (11)	196 (11)	116 (11)	275 (11)	6,798 (11)
Former	1,314 (113)	209 (113)	186 (113)	116 (113)	247 (113)	6,597 (104)
Current	1,160 (48)	216 (48)	188 (48)	115 (48)	303 (48)	5,970 (46)
Unknown	1,193 (14)	205 (14)	180 (14)	112 (14)	277 (14)	6,798 (11)
*p* - value	0.996	0.409	0.550	0.848	0.650	0.051
**Gender,** Mean (N)						
Male	1,161 (100)	213 (100)	186 (100)	114 (100)	256 (100)	6,071 (91)
Female	1,238 (86)	204 (86)	188 (86)	117 (86)	270 (86)	6,551 (82)
*p* - value	0.485	0.475	0.637	0.515	0.832	0.49

* The maximum range of AQUA scores in version 2.3.4.1 is 0–33,333.

We found no significant (*p*>0.05) associations of the *in situ* levels of these proteins with the clinical parameters tumor histology (categorized as adenocarcinoma, squamous cell carcinoma, or large cell carcinoma), tumor size (categorized as *≤*2 cm, >2 to *≤*3 cm, >3 to *≤*5 cm, >5 to *≤*7 cm, and >7 cm), smoking status (categorized a never smoker if the patient had smoked less than 100 cigarettes during life time, former smokers if the patient had quit for at least 1 year, and current smoker for all others), and gender (table 3).

## Discussion

Systemic therapy, targeted at disrupting DNA synthesis, continues to be the mainstay of anti-cancer therapy, and it is likely to remain a cornerstone for years to come. Antimetabolites are a class of DNA-targeted agents, and gemcitabine is a widely used class representative for treatment of solid malignancies [1], [2], [3], [4]. RRM1 is a validated, predictive marker of gemcitabine efficacy [11], [12], [13], [14], which provides an opportunity for the identification of molecules that critically interact with RRM1 to maintain cell proliferation and viability [16], [17], [18]. To improve *de novo* therapeutic efficacy and attenuate the emergence of resistance, kinases are particularly attractive molecules because of their role in proliferation and viability and because many are druggable targets with available inhibitors in clinical development [25].

Synthetic lethality screening is a well-suited method for the identification of molecules that critically interact to maintain viability [26]. Originally, synthetic lethality was the identification of two genes whose simultaneous loss-of-function resulted in cell death in yeast as a model organism, while loss of either gene alone was compatible with life [27]. However, in an expanded application, two molecules can be referred to as “synthetically lethal” if functional attenuation of two molecules synergistically impacts on a desired outcome. We thus deployed this method to assess the impact of attenuation by siRNA technology of a series of kinases on gemcitabine efficacy in the context of RRM1 levels.

We deployed a stringent algorithm that consisted of multiple stages of siRNA knockdowns in a series of gemcitabine-resistant cell line models, including cell lines naturally resistant, with gemcitabine-induced resistance, and with genetically-induced resistance, to identify the most promising kinase target. Among the 87 kinases screened, we identified CHK1 as the lead kinase with consistent interaction with RRM1-dependent gemcitabine efficacy, and this synthetic lethality interaction was validated using AZD7762, a small-molecule ATP-competitive inhibitor of CHK1 activation [20].

Our identification of CHK1 as a potent modulator of RRM1-dependent gemcitabine efficacy is not surprising. The serine/threonine kinase CHK1 is a key effector in response to DNA damage, which triggers arrest of G2 cell cycle progression and facilitates DNA damage repair [28], [29]. It becomes activated by the phosphatidylinositol 3-kinase-like serine/threonine kinases ATR (ataxia telangiectasia response) and ATM (ataxia telangiectasia mutated) through phosphorylation of Ser-317 and Ser-345 [28] followed by autophosphorylation of Ser-296 [30]. It has recently been identified as a lead therapeutic target in neuroblastoma [31], and prior work by the AstraZeneca research and development team identified CHK1 inhibition by AZD7762 as contributing to efficacy of DNA-damaging agents [20].

We observed a consistent decrease in RRM1 expression levels with CHK1 knockdown, which may partially explain the improved gemcitabine efficacy. However, we also observed a decrease in RRM2 levels, and published reports had demonstrated that gemcitabine efficacy is unrelated to RRM2 levels [9], [10], [15]. However, other mechanisms may also contribute to the observed CHK1 interaction with RRM1-dependent gemcitabine efficacy. Our prior work had identified delayed G2 cell cycle progression as a consequence of RRM1 overexpression [18], and the results presented here demonstrate a higher degree of synergism between CHK1 inhibition and RRM1 in cells with high levels of RRM1 expression compared to those with low RRM1 expression.

We had previously identified p53 inactivation as contributing to gemcitabine resistance in a panel of 26 NSCLC cell lines [19]. In addition, p53 knockdown in p53 expressing cell lines increased and p53 restoration in p53-null cells decreased gemcitabine resistance. Notably, all four cell lines with naturally occurring gemcitabine resistance (IC50 above 1 mM) used here have non-functional p53 alterations (H125, truncation N239*; H650, missense K164N; H1648, truncation D42*; H2122, missense C176F and an additional missense Q16L with no predicted functional impact; http://www-p53.iarc.fr/MutationValidation.asp?Mutant=K164N), and we observed an up to 50-fold improvement in gemcitabine efficacy with CHK1 knockdown. These results are consistent with earlier reports suggesting that abrogation of the G2 checkpoint may be particularly effective in cells with impaired p53 function [32], [33], [34].

In conclusion, we identified CHK1 inhibition as a major modulator of RRM1-dependent gemcitabine efficacy using a synthetic lethality screen with 87 protein kinases. The impact of CHK1 inhibition is particularly pronounced in cells resistant to gemcitabine as a result of high RRM1 expression levels. This implies that the combination of a CHK1 inhibitor and gemcitabine is likely to be most effective in patients with high tumoral levels of RRM1. Our quantitative *in situ* determination of RRM1 and total CHK1 protein levels demonstrated that CHK1 levels tend to decrease as RRM1 levels increase, which suggests that CHK1 inhibition may be best achieved in patients with RRM1-dependent gemcitabine resistance. Clinical trials utilizing both agents may provide an opportunity to assess this interaction in patients with solid malignancies and may afford the opportunity for patient stratification based on RRM1 levels.

## Materials and Methods

### Cell Lines, Culture Conditions, and Drugs

The human NSCLC cell lines H23, H125, H650, H1299, H1648, and H2122 were purchased from the American Type Culture Collection. They were maintained in RPMI 1640 supplemented with 10% fetal bovine serum, 2 mmol/L L-glutamine, penicillin (25 units/mL), and streptomycin (25 µg/mL) in a humidified atmosphere with 5% CO_2_ at 37°C. MCF7 human mammary adenocarcinoma cells were maintained in MEM-α supplemented with 10% fetal bovine serum, penicillin/streptomycin, non-essential aminoacids (0.1 mM), sodium pyruvate (1 mM), sodium bicarbonate (1.5 g/L), and bovine pancreatic insulin (0.01 mg/mL). The two human cell line model systems derived from wild-type (wt) H23 and MCF7 with stably increased (H23-R1 and MCF7-R1) and decreased RRM1 expression (H23-shR1 and MCF7-shR1) as well as control vector transfection (H23-Ct and MCF7-Ct) have been previously described [19]. Authenticity of cell lines was confirmed by DNA finger printing.

Gemcitabine (Eli Lilly) resistance was generated by treating H23 and H1299 cells with 1 nM gemcitabine for two weeks. Surviving cells were then exposed to increasing doses until cells survived drug concentrations ten-fold higher than the original gemcitabine IC50 (8 nM for H23, 24 nM for H1299 [19]). Clonal sublines of resistant cells were generated from single colonies (H23-G-C8 and C23; H1299-G-C2 and C18), and resistance was confirmed using a cell viability assay as described below. RRM1 expression was assessed by Western blotting.

Thiophene carboxamide urea (AZD7762), an ATP-competitive inhibitor of checkpoint kinase 1 (CHK1), and vandetanib (AZD6474), an inhibitor of KDR, EGFR, and RET activation, were obtained from Synthesis Med Chem Pty Ltd, Australia. They were dissolved in 0.1% DMSO, and all experiments using these agents included controls with 0.1% DMSO alone.

### Cell Viability Assay and Synthetic Lethality Screening

Cells were plated in 96-well plates for 16 h and then treated with increasing concentrations of gemcitabine (10 pM–100 mM) for 4 to 7 days in the presence or absence of 150 nM or 300 nM AZD7762. Dose response was determined using a luminescent cell viability assay (CellTiter-Glo, Promega, Madison WI) according to the manufacturer's instructions. Assay data were transferred to a spreadsheet program to calculate the percent viability relative to the replicate control cell wells that did not receive drug. The half-maximal inhibitory concentrations; i.e., IC_50_ values, were calculated using SigmaPlot dose-response curves (Systat Software).

For drug combination experiments, drugs were used at fixed dose ratios (i.e. 50∶1, 1∶10, 1∶250), and results were analyzed for synergistic, additive, or antagonistic effects using the combination index (CI) method [21]. Briefly, the dose-effect curve for each drug alone was determined based on experimental observations using the median-effect principle and compared to the effect achieved with a combination of two drugs to derive a CI value. The method involves plotting dose-effect curves for each agent and their combination using the median-effect equation: fa/fu = (D/Dm) m, where D is the dose of the drug, Dm the dose required for a 50% effect (equivalent to IC50), fa and fu the affected and unaffected fractions (fa = 1-fu), and m the exponent signifying the sigmoidicity of the dose-effect curve. The computer software XLfit was used to calculate the values of Dm and m. The CI used for the analysis of the drug combinations was determined by the isobologram equation for mutually nonexclusive drugs that have different modes of action: CI = (D)_1_/(Dx)_1_+(D)_2_/(Dx)_2_+(D)_1_(D)_2_/(Dx)_1_(Dx)_2_, where (Dx)_1_ and (Dx)_2_ in the denominators are the doses (or concentrations) for drug 1 and drug 2 alone that gives x% inhibition, whereas (D)_1_ and (D)_2_ in the numerators are the doses of drug 1 and drug 2 in combination that also inhibited x% (i.e., isoeffective). Combination indices CI<1, CI = 1, and CI>1 indicate synergism, additive effects, and antagonism, respectively.

Gene specific knockdowns were done using the Dharmacon siGENOME SMARTpool and ON-TARGET*plus* SMARTpool libraries (Dharmacon RNAi Technologies). Non-targeting siRNA was used as a control in all experiments. Transfections were done with Lipofectamine RNAiMAX (Invitrogen) following the manufacturer’s instructions using 30 nM siRNA concentrations unless indicated otherwise. Drugs were added 24 h after siRNA transfection.

### RNA Isolation, cDNA Synthesis, and Quantitative Real-time PCR

Total RNA was isolated from cells 24 h after siRNA transfection, unless indicated otherwise, using a commercially available method (RNeasy, Qiagen) and reverse transcribed to generate cDNA (QuantiTect, Qiagen). A fluorescence-based real-time detection method (Taqman, Applied Biosystems) was used for quantification of gene expression. The primers and probes used for CHK1 and 18s-rRNA were purchased from ABI, and those for RRM1 and RRM2 have been published [19]. Reaction conditions and gene expression value calculations using 18s-rRNA as reference gene were as previously described [19].

### Western Blot Analysis

Cells were lysed (50 mM Tris-HCl pH 7.5, 150 mM NaCl, 0.5% NP-40, 10 mM glycerol, 5 mM EDTA, 0.5% sodium deoxycholate) in the presence of protease and phosphatase inhibitors (1 mM phenylmethylsulfonyl fluoride, 1 µg/mL aprotinin, 1 µg/mL leupeptin, 1 mM sodium orthovanadate). Proteins were separated in 8 to 10% SDS-polyacrylamide gels and blotted onto nitrocellulose membranes (Biorad). Filters were incubated with polyclonal antisera or monoclonal antibodies against β-actin, RRM1, RRM2, PTEN, MCM7 (Santa Cruz Biotechnology), and Chk1 (Cell Signaling).

### CHK1 and RRM1 *in situ* Protein Analysis

The use of specimens from human subjects complied with the Helsinki Declaration and was approved by the institutional review board of Wayne State University. Tissue microarrays (TMAs) consisting of three 0.6 mm in diameter replicates of surgically resected, formalin-fixed and paraffin embedded (FFPE), tumor specimens from patients with NSCLC had been constructed as previously described [23]. Sections of 5 µm thickness were transferred onto adhesive-coated glass slides, deparaffinized, and processed for antigen retrieval. The reagents and dilutions used for target detection were NBP1-61573 (rabbit polyclonal, Novus Biologicals), 1∶50 for total CHK1; NBP1-60797 (rabbit polyclonal, Novus Biologicals), 1∶25 for pCHK1(S280); NBP1-40633 (rabbit monoclonal, Novus Biologicals), 1∶25 for pCHK1(S296); NB100-92499 (rabbit polyclonal, Novus Biologicals), 1∶25 for pCHK1(S317); ab47318 (rabbit polyclonal, Abcam), 1∶100 for pCHK1(S345); and R1-E4138-C42 (rabbit monoclonal, generated against full-length RRM1), 1∶1 for RRM1. For identification of carcinomatous cells, an antibody/antiserum to cytokeratin was used (murine anti-human pancytokeratin AE1/AE3, 1∶200, #M3515, Dako Cytomation; rabbit anti-human pancytokeratin AE1/AE3, 1∶200, #Z0622, Dako Cytomation). Slides were washed and incubated with two different secondary antibodies for 1 hr (Envision® labeled polymer-HRP anti-rabbit, # K4011, or Envision® labeled polymer-HRP anti-mouse, # K4007, specific to the primary antibody/antiserum used for target protein detection, 1∶200; Alexa 555 goat anti-mouse, #A21424, or goat anti-rabbit, #A21429, based on the source of the anti-cytokeratin antibody/antiserum, 1∶200, Dako Cytomation). For fluorescence amplification, slides were exposed to Cy5-Tyramide (1∶50) for 10 min at room temperature and mounted with Prolong Gold antifade reagent with DAPI. The final slides were scanned with SpotGrabber, image data were captured with a digital camera and fluorescence microscope, and *in situ* detection of targets and quantification of expression levels in the nuclear compartment was done by AQUA (PM-2000, version 2.3.4.1, HistoRx, New Haven, Connecticut).

### Statistical Analysis

The average value of three TMA spot replicates was calculated for each patient and target.

Associations among parameters with continuous values were calculated using the Spearman rank correlation coefficient. Associations between continuous and categorical values were calculated using the Wilcoxon rank-sum test for variables with two categories and the Kruskal-Wallis test for variables with three or more categories.
